# Phosphoinositide 3-kinase-delta could be a biomarker for eosinophilic nasal polyps

**DOI:** 10.1038/s41598-018-34345-3

**Published:** 2018-10-30

**Authors:** Jong Seung Kim, Jae Seok Jeong, Kyung Bae Lee, So Ri Kim, Yeong Hun Choe, Sam Hyun Kwon, Seong Ho Cho, Yong Chul Lee

**Affiliations:** 1 Department of Otorhinolaryngology-Head and Neck Surgery, Chonbuk National University Medical School, Jeonju, South Korea; 2 Department of Internal Medicine, Research Center for Pulmonary Disorders, Chonbuk National University Medical School, Jeonju, South Korea; 3 Research Institute of Clinical Medicine, Chonbuk National University-Biomedical Research Institute, Chonbuk National University Hospital, Jeonju, South Korea; 4 Division of Allergy and Immunology, Internal Medicine, Morsani College of Medicine, University of South Florida, Tampa, FL USA

## Abstract

Nasal polyps (NP) cause diverse clinical symptoms of chronic rhinosinusitis (CRS). Chronic inflammation of sinonasal mucosa is known to be crucial in NP formation. We aimed to define the implications of phosphoinositide 3-kinase (PI3K)-δ in nasal inflammation associated with NP by analyzing NP tissue obtained from CRS patients. Results showed that expression of p110δ, a regulatory subunit of PI3K-δ, in NP tissue was increased compared to control tissue. Increased p110δ expression was closely correlated with more severe CRS features. Interestingly, p110δ expression was increased in eosinophilic NP, which are closely related to more complicated clinical courses of the disease. Furthermore, CRS patients possessing NP with higher p110δ expression displayed more eosinophils in NP tissue and blood, higher levels of IL-5 in NP tissue, and more severe features of the disease. Therefore, PI3K-δ may contribute to the formation of NP, especially eosinophilic NP associated with more severe clinical presentations and radiological features.

## Introduction

Nasal polyps (NP) arising from the sinonasal area are almost always associated with chronic rhinosinusitis (CRS) and can cause a wide array of clinical symptoms and disturbance of daily life, thereby significantly impacting on quality of life and medical expenditure related to management of the disease^1^. In particular, the recurrence rate of NP in CRS has been reported to be high, up to over 50%, despite intensive treatment including surgery^2^. Thus, it has been widely accepted that the presence of NP is a key factor in dividing CRS into two different clinical phenotypes. Previous studies have suggested that CRS associated with NP predominantly manifests eosinophil-dominant T helper type 2 cell (T_H_2)-associated inflammation, in which interleukin (IL)−5 is crucially implicated in the pathogenesis of the disease^3^. This type of CRS is known to be more resistant to medical and surgical treatment and related to frequent recurrence, compared to CRS without NP^4^. However, this simplified phenotypical classification based on the presence of NP does not seem to correctly reflect the underlying pathobiologic process. Indeed, the need for more advanced subtyping approaches that incorporate specific biological mechanisms (*i.e*. endotypes) of CRS is urgent^5^.

Through being associated with a broad range of cell surface receptors, the phosphoinositide 3-kinases (PI3Ks) in human cells, which catalyze the phosphorylation of membrane inositol lipids to produce a second messenger phospholipid (*i.e*. phosphatidylinositol-3,4,5-trisphosphate) and subsequent activation of effector proteins such as Akt^6^, have been known to play key roles in diverse cellular processes including cell growth and proliferation, migration, metabolism, and immune responses. They exist as heterodimers comprising a catalytic p110 subunit (α, β, γ, and δ) with a particular regulatory subunit. In view of immune and inflammatory processes involving numerous cell types, the p110δ isoform has been regarded as a crucial target for drug-mediated PI3K isoform inhibition due to its enriched expression in immune cells, whereas the p110α and p110β isoforms are ubiquitously expressed. Immunologic roles of PI3K-δ involve T- and B-cell activation^7^, mast cell degranulation^8^, and the recruitment of eosinophils and neutrophils into inflamed tissue^9,10^. In particular, modulation of this signaling pathway has been shown to be effective for ameliorating T_H_2-associated eosinophil-dominant lung inflammation including corticosteroid-resistant severe forms of the disease^11–13^.

It is not known exactly how NP develops, but chronic inflammation on sinonasal mucosal homeostasis owing to various inflammatory stimuli have been suggested to be important^14^. Furthermore, several inflammatory mediators and signaling pathways may be involved in the formation of NP^15^. However, a potential role of PI3K-δ signaling in the formation of NP and associated inflammation in the nasal cavity has not been characterized. Based on this knowledge, in this study, we aimed to define the possible implications of PI3K-δ in nasal inflammation associated with NP by analyzing NP tissue obtained from patients with CRS. Furthermore, we investigated whether PI3K-δ activation could identify a particular phenotype of CRS having distinct clinical characteristics through assessing the levels of immune mediators in NP tissue and calculating the endoscopic, radiographic, and symptomatic scores in the patients.

## Methods

### Patients and tissue preparation

A total of 43 patients were enrolled in this study. Among them, 33 subjects had NP and concurrent CRS. We also obtained inferior turbinate tissues from 10 control subjects without CRS who underwent other rhinological surgical procedures (*e.g*. septoplasty). The NP tissues were cut into two pieces. Half of the samples were immediately frozen to quantify the expression of PI3K-δ by Western blotting, while the other half were fixed with 4% paraformaldehyde and subsequently used for immunofluorescence staining. All experimental protocols regarding human tissues were approved by the Institutional Review Board of the Biomedical Research Institute of Chonbuk National University Hospital (IRB file No. 2017-01-019). We confirm that all methods were performed in accordance with the relevant guidelines and regulations. Written informed consents were obtained from all participants. The clinical characteristics of the patients are described in Table 1. To be eligible, participants had to meet all of the following criteria at enrollment: (1) older than 18 years of age; (2) provide written informed consent; (3) inflammation of the nose and the paranasal sinuses for longer than 12 weeks characterized by two or more symptoms of nasal obstruction, nasal discharge, facial pain, or loss of smell and endoscopic sign of nasal polyps, mucopurulent discharge, or edema in the middle meatus that could not be controlled by antibiotic therapy. Patients meeting the following criteria were excluded: (1) less than 18 years of age; (2) did not provide informed consent; (3) prior treatment with antibiotics, systemic or topical corticosteroids, or other immune modulating drugs in the 4-week period before surgery, (4) state of pregnancy; (5) had a history of previous sinus surgery; (6) having unilateral rhinosinusitis, cystic fibrosis, or immotile ciliary disease.

**Table 1:** Demographic and clinical characteristics of 33 patients with NP and concurrent CRS and 10 control subjects without CRS.

	Control	NEP	EP
N	10	15	18
M/F	8/2	5/10	14/4
Age (years): mean (SD)	37.1 (17.7)	54.7 (19.1)	46.9 (13.8)
Asthma (%)	0 (0)	0 (0)	1 (5.3)
Aspirin sensitivity	0 (0)	0 (0)	0 (0)
Other past history	0	DM 2, gastric cancer 1	DM 1, lung cancer 1
Tissue used	IT	Nasal polyps	Nasal polyps
SNOT-22 scores: mean, (SD)	15.6 (6.4)	37.3 (13.0)	48.7 (24.2)

N, number; NP, nasal polyps; CRS, chronic rhinosinusitis; NEP, non-eosinophilic polyps; EP, eosinophilic polyps; M, male; F, female; IT, inferior turbinate; DM, diabetes mellitus; SD, standard deviation.

### Subdivision of CRS patients according to p110δ expression

Quantitative measurements of p110δ expression in NP tissue were performed using densitometric analyses of Western blotting data. In short, the results were expressed as the relative ratio of p110δ protein to actin. The relative ratio of the target protein in the control group was arbitrarily taken to be 1. Then, we subdivided these CRS patients according to the extent of p110δ expression.

A recent report has demonstrated that peripheral blood count has a profound effect on the division of non-eosinophilic nasal polyps (NEP) and eosinophilic nasal polyps (EP)^16^. The cut-off value for the p110δ level that affects the NEP and EP was obtained by the decision tree method and non-hierarchical cluster method^17–22^. We first performed cluster analysis using all the data including p110δ level and blood eosinophil. We used K-means cluster analysis using non-hierarchical cluster method in R software. K-means clustering is a popular method for cluster analysis in data mining. It can be used to partition the input data set into k clusters^23–25^.

Among the decision tree methods, we used two packages, “tree” and “rpart” in R statistical software, version 3.3.1, to determine the cut-off value for p110δ, and the results of both packages matched 2.833-fold. As a result, when there was more than 2.833-fold of p110δ expression in NP tissues compared to those of control subjects, those patients were regarded as having NP with higher p110δ expression (NP p110δ^hi^). Likewise, when there was less than 2.833-fold of p110δ expression in NP tissues compared to those of control subjects, those patients were regarded as having NP with lower p110δ expression (NP p110δ^lo^). We have validated our model using both packages, and have optimized the decision tree through pruning to compensate for the disadvantages of overmatched convergence in both packages^26^.

### Immunofluorescence staining for p110δ and eosinophil cationic protein (ECP)

Paraffin-embedded human NP tissue sections were deparaffinized and hydrated. The sections were fixed with ice-cold methanol and permeabilized in phosphate-buffered saline (PBS) containing 0.25% Triton X-100 for 10 minutes at room temperature and washed 3 times with PBS. Subsequently, nonspecific bindings were blocked with 1% bovine serum albumin (BSA) (Sigma–Aldrich, St. Louis, MO, USA) in PBS containing 0.05% Tween 20 for 1 hour. Specimens were then incubated in a humidified chamber for 2 hours at room temperature with an anti-p110δ antibody (Ab) (Santa Cruz Biotechnology, Dallas, TX, USA) and anti-RNASE3 (ECP) Ab (Thermo Fisher Scientific Inc., Waltham, MA, USA). For the detection of each binding Ab to p110δ and ECP, Alexa Fluor 488 (green)-labeled goat anti-mouse IgG (Thermo Fisher Scientific Inc.) and Alexa Fluor 546 (red)-labeled goat anti-rabbit IgG (Thermo Fisher Scientific Inc.) in 1% BSA, respectively, were loaded for 1 hour at room temperature in the dark. After the specimens were washed, nuclei were stained with 4′,6-diamidino-2-phenylindole (DAPI) (Thermo Fisher Scientific Inc). Stained cells were mounted on slides with fluorescent mounting medium (Golden Bridge International, Mukilteo, WA, USA) and then visualized with a confocal microscope (Zeiss LSM510 Meta, Carl Zeiss, Jena, Germany). Quantification of immunofluorescence intensities of p110δ in confocal microscope were performed using ImageJ.

### Western blot analysis

Human NP tissues were homogenized in the presence of protease inhibitor cocktail (Sigma–Aldrich), and protein concentrations were determined using Bradford reagent (Bio-Rad Laboratories, Hercules, CA, USA). Samples were loaded onto a SDS–PAGE gel. After electrophoresis at 120 V for 90 minutes, proteins were transferred to polyvinylidene difluoride (PVDF) membranes (Bio-Rad Laboratories) at 250 mA for 90 minutes by a wet transfer method. Nonspecific sites were blocked with 5% non-fat dry milk in Tris-buffered saline Tween 20 (25 mmol/L Tris pH 7.5, 150 mmol/L NaCl, 0.1% Tween 20) for 1 hour, and the blots were then incubated overnight at 4 °C with an anti-p110δ Ab (Santa Cruz Biotechnology), anti-phospho-AKT Ab (R&D Systems, Minneapolis, MN, USA), anti-AKT Ab (Cell Signaling Technologies, Danvers, MA, USA), anti-IL-5 Ab (Santa Cruz Biotechnology), and anti-actin Ab (Sigma–Aldrich). Anti-rabbit or anti-mouse (Cell Signaling Technologies) horseradish peroxidase (HRP)-conjugated IgG was used to detect binding of Abs. The binding of the specific Ab was visualized using Fuji film LAS-3000 (Fuji film) after treating with enhanced chemiluminescence system reagents (GE Healthcare, Little Chalfont, Buckinghamshire, UK). For the quantification of specific bands using the Multi Gauge program (version 3.0, Fuji film), a square with the same size was drawn around each band to measure the density and then the value was adjusted by the density of the background near that band. The results of densitometric analysis were expressed as the relative ratio of the target protein to reference protein. The relative ratio of the target protein in the control group was arbitrarily taken to be 1.

### Statistical analysis

We used SPSS statistical software (version 19.0, IBM Corp., Armonk, NY, USA) and R 3.3.1. Data were expressed as mean ± SD. Statistical comparisons were performed using one-way ANOVA followed by the Scheffe’s test (^*^P < 0.05, ^**^P < 0.01 or ^***^P < 0.001). The Mann–Whitney *U*-test was used to compare the two groups.

## Results

**Figure 1:**
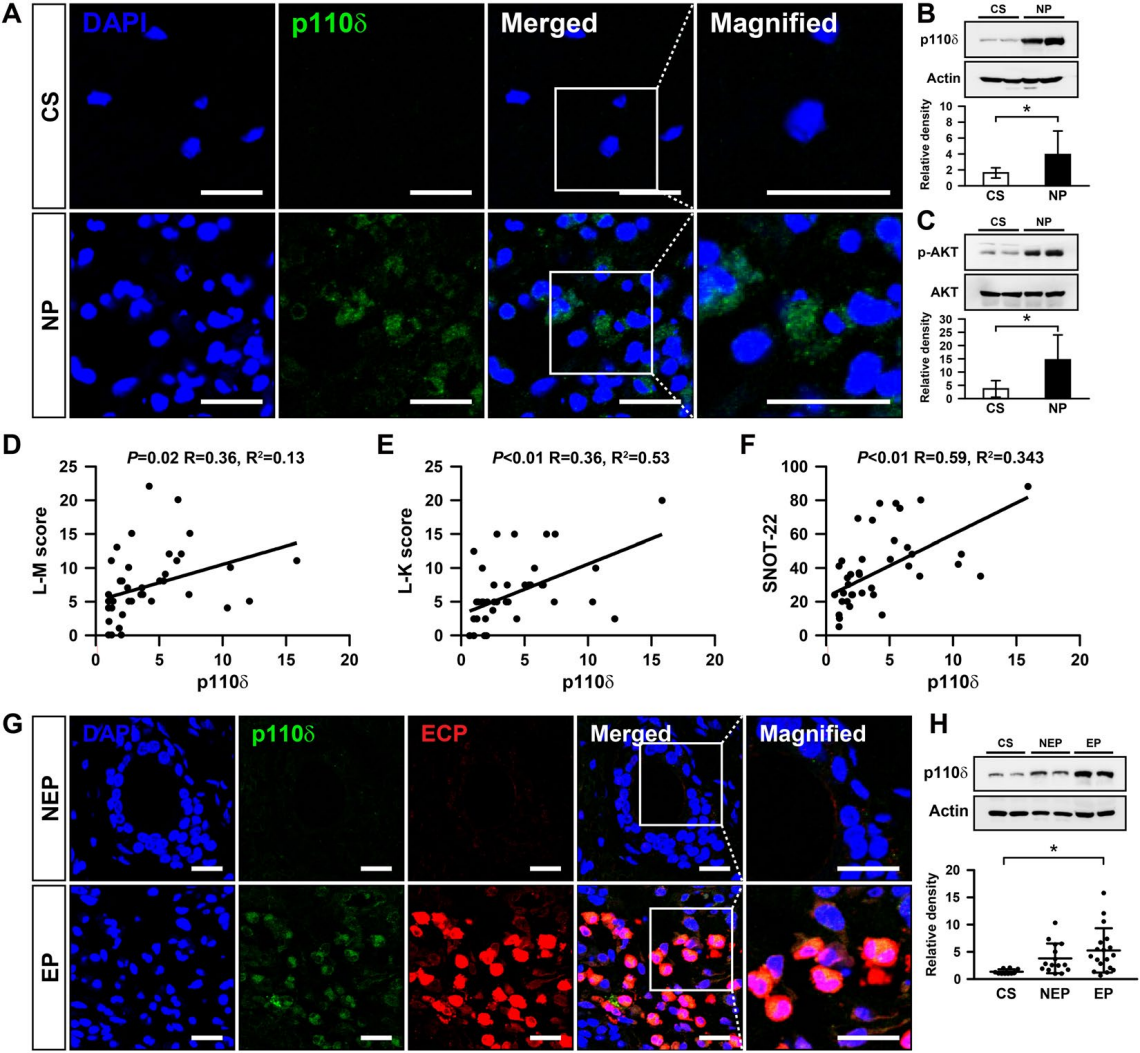
(**A**) Representative confocal images of nasal polyp (NP) tissue demonstrate the localization of a catalytic p110 subunit (p110δ) of PI3K-δ (green) from chronic rhinosinusitis (CRS) patients. Immunofluorescence intensities of p110δ in inferior turbinate tissue from control subjects (CS) are shown as control. 4′,6-diamidino-2-phenylindole (DAPI) stain was used for nuclear localization. Bars indicate 20 μm. (**B**) Representative immunoblots and densitometric analyses of p110δ in NP tissues from CRS patients or in inferior turbinate tissues from CS. Bars represent mean ± SD. ^*^P < 0.05 versus CS. (**C**) Representative immunoblots of phosphorylated (p)-AKT and total AKT and densitometric analyses of p-AKT in NP tissues from CRS patients or inferior turbinate tissues from CS. Bars represent mean ± SD. ^*^P < 0.05 versus CS. Correlation between p110δ expression and the Lund-Mackay (L-M) computed tomography score (**D**), the Lund-Kennedy (L-K) endoscopic score (**E**), and the Sino-Nasal Outcome Test-22 [SNOT-22] questionnaire score (**F**) in CRS patients. (**G**) Representative confocal images of NP tissues show the localization of p110δ and eosinophil cationic protein (ECP) in NP tissues from CRS patients with eosinophilic NP (EP) or non-eosinophilic NP (NEP). Bars indicate 20 μm. (**H**) Representative immunoblots and densitometric analyses of p110δ in NP tissues from CRS patients with EP or NEP or in inferior turbinate tissues from CS. ^*^P < 0.05 versus CS.

To investigate whether PI3K-δ is involved in the formation of NP, we first checked the protein expression of the catalytic subunit of PI3K-δ (p110δ) in NP tissue. Confocal microscopic analysis showed that immunofluorescence intensities of p110δ were remarkably increased in NP tissue compared to those of control tissue (Fig. 1A and Supplemental Fig. 1). In the same context, Western blot analysis demonstrated that protein levels of p110δ in NP tissue were significantly increased compared to controls (Fig. 1B). Protein levels of phosphorylated AKT, a key downstream mediator of PI3K-δ signaling, were also notably elevated in NP tissue (Fig. 1C). Furthermore, increased production of p110δ protein in NP tissue was closely correlated with a much higher radiographic score (by the Lund-Mackay score, Fig. 1D), endoscopic score (by the Lund-Kennedy endoscopic scoring system, Fig. 1E), and symptom score (by the Sino-Nasal Outcome Test-22 [SNOT-22] questionnaire, Fig. 1F) in NP patients. These findings suggest that PI3K-δ may influence the formation of NP having much higher clinical and radiological scores.

We also investigated whether PI3K-δ contributes to eosinophilic inflammation in NP of CRS patients. The results showed that immunofluorescence intensities of p110δ were dramatically increased in eosinophilic NP (EP; NP patients with blood eosinophils $\ge$200 cells/μL) compared to those of non-eosinophilic NP (NEP; NP patients with blood eosinophils $<$200 cells/μL) (Fig. 1G and Supplemental Fig. 2). Furthermore, immunofluorescence intensities of eosinophil cationic protein (ECP), a marker protein for eosinophils, were notably increased in EP compared to those in NEP, as expected, and we observed prominent co-localization of p110δ and ECP in EP (Fig. 1G). In addition, Western blot analysis showed a tendency toward an increase in the levels of p110δ protein in EP compared to those in NEP (Fig. 1H). These findings suggest that PI3K-δ may be involved in the formation of NP, particularly eosinophilic NP that are closely related to more complicated clinical courses of the disease.

**Figure 2:**
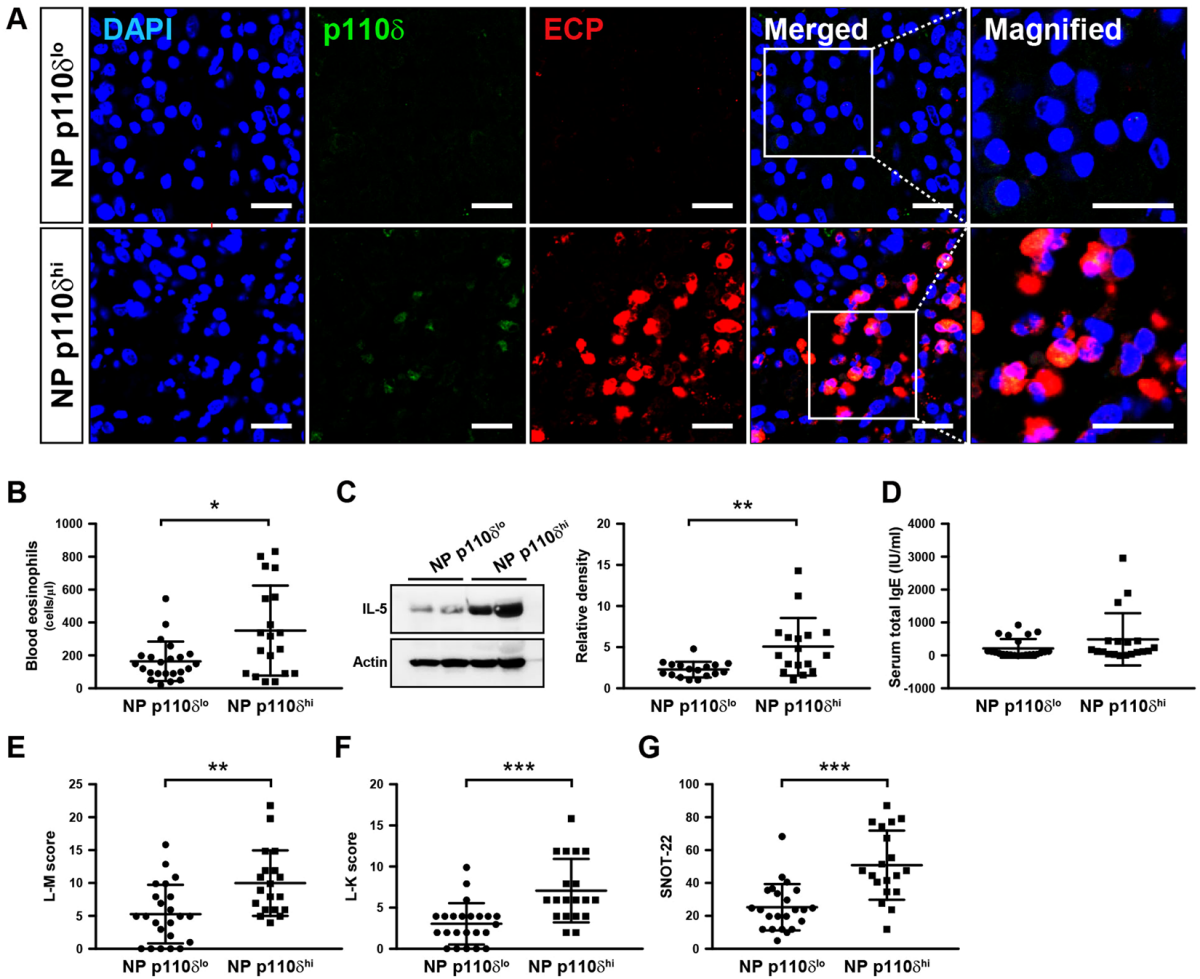
(**A**) Representative confocal images of nasal polyp (NP) tissue show the localization of p110δ and ECP in NP tissue with higher p110δ expression (NP p110δ^hi^) or NP with lower p110δ expression (NP p110δ^lo^). Bars indicate 20 μm. (**B**) Blood counts of eosinophils in CRS patients with NP p110δ^hi^ or NP p110δ^lo^. ^*^P < 0.05 versus CRS patients with NP p110δ^lo^. (**C**) Representative immunoblots and densitometric analyses of IL-5 protein in NP tissue from CRS patients with NP p110δ^hi^ or NP p110δ^lo^. ^**^P < 0.01 versus CRS patients with NP p110δ^lo^ (**D**) Levels of serum total Ig-E in CRS patients with NP p110δ^hi^ or NP p110δ^lo^. (**E**–**G**) Calculated L-M score (**E**), L-K scores (**F**), and SNOT-22 scores (**G**) in CRS patients with NP p110δ^hi^ or NP p110δ^lo^. ^**^P < 0.01 or ^***^P < 0.001 versus CRS patients with NP p110δ^lo^.

Lastly, to investigate whether PI3K-δ activation could identify a particular subtype of CRS having distinct clinical behavior, we subdivided the CRS patients according to the extent of p110δ expression (*i.e*. when there was more than 2.833-fold of p110δ expression in NP tissue compared to those of control subjects, those patients were regarded as having NP with higher p110δ expression [NP p110δ^hi^]), and then analyzed the related immune mediators and clinical parameters of those patients. The data showed that immunofluorescence intensities of p110δ and ECP and their co-localization were substantially increased in NP p110δ^hi^ compared to those in NP with lower p110δ expression (NP p110δ^lo^) (Fig. 2A). Blood counts of eosinophils in patients with NP p110δ^hi^ were also significantly increased compared to those with NP p110δ^lo^ (Fig. 2B). Tissue levels of IL-5 protein were notably increased in NP p110δ^hi^ (Fig. 2C). However, there was no statistically significant difference in serum total Ig-E levels between the two groups (Fig. 2D), suggesting that the adaptive immune response involving Ig-E may not be closely associated with eosinophilic CRS mediated by PI3K-δ. Furthermore, patients possessing NP p110δ^hi^ had much higher radiologic and endoscopic scores (Fig. 2E,F). Lastly, patients with NP p110δ^hi^ displayed much higher sinonasal symptom scores compared to those with NP p110δ^lo^ (Fig. 2G).

## Discussion

In this study, the protein expression of p110δ protein and AKT in NP tissue was significantly increased compared to that in controls. Interestingly, increased production of p110δ protein in NP tissues was closely correlated with much higher radiographic, endoscopic, and symptom scores in CRS patients. Furthermore, we observed a tendency toward an increase in the levels of p110δ protein in EP compared to those in NEP, suggesting that the PI3K-δ pathway may be involved in the formation of NP, particularly eosinophilic NP that are closely related to more complicated clinical courses of the disease. This was further verified by the finding that CRS patients possessing NP with higher p110δ expression displayed more eosinophils in NP tissue and blood, higher levels of IL-5 in NP tissue, and more severe features of underlying CRS compared to CRS patients possessing NP with lower p110δ expression.

Chronic inflammation has been reported to be important in the development of NP in CRS patients, and several inflammatory mediators and signaling pathways have been reported to be involved in the formation of NP^15^. In particular, sinonasal eosinophilic inflammation associated with upregulation of T_H_2-mediated immune response has been regarded as a crucial feature of the disease^4^. In this process, the maturation of eosinophils occurs in bone marrow under the influence of IL-5 and they are released into the blood circulation. Then, type 2 inflammatory milieus involving various cytokines (e.g. IL-4 and IL-13) and chemokines are produced by surrounding tissues to control transmigration and enhanced survival of eosinophils in the nasal cavity. In this view, several biomarkers related to type 2 immune response have been suggested as potential predictors for the presence of NP in CRS patients. For example, serum and tissue expression of periostin (a well-known marker for eosinophil/type 2 inflammation in bronchial asthma since the initial report on the relevance of its gene expression in airway epithelial cells to T_H_2-driven inflammation^27^) has also been reported to be a predictor for the presence and postoperative recurrence of NPs as well as the comorbid NPs in patients with bronchial asthma^28–30^. However, not enough is known regarding the exact role of this matricellular protein in the pathophysiology of CRS as well as its potential implications as an ideal biomarker for the development of novel therapeutic drugs^31^.

The PI3K-δ signaling pathway is likely to be involved in many aspects of NP development particularly in association with eosinophilic functions, given its well-known roles in the pathogenesis of allergic lung inflammatory disorders. We previously showed that PI3K-δ blockade significantly lowered the allergen-induced increased levels of IL-5, the most important cytokine for maturation, growth, differentiation, and survival of eosinophils, in the lung^12,13^. In addition, inhibition of PI3K-δ significantly lowered the allergen-induced increases of leukotriene C4, eotaxin, RANTES, ICAM-1, VCAM-1, IL-4, IL-5, and IL-13 in the lung, all of which orchestrate selective tissue recruitment/transmigration of eosinophils^13^. PI3K-δ is also likely to play key roles in generating proper effector functions of eosinophils involving the release of diverse eosinophil-derived mediators (*e.g*. granular proteins, lipid mediators, cytokines/chemokines, enzymes, growth factors, and oxidative products) partly through innate receptor-mediated generation of reactive oxygen species (ROS) and pro-inflammatory cytokines^11,12^. Several indirect modulatory effects of PI3K-δ on eosinophilic inflammation also seem to exist. PI3K-δ has been reported to play an important role in the adaptive immune response involving T- and B-cells^7,32^, influencing adaptive T_H_2-mediated eosinophilic inflammation. Furthermore, we have reported the crucial modulatory roles of PI3K-δ in the innate immune response at the airway epithelium partly via regulation of epithelial generation of mitochondrial ROS^11,12^, which are known to be essential for T_H_2 cell-associated eosinophilic inflammation in airways^33^. More recently, reduction of chemokines involved in allergen-induced recruitment of T_H_2 lymphocytes (namely, CCL17 and CCL22) by a potent small molecular inhibitor of PI3K-δ has been demonstrated in a phase I clinical study in patients with allergic rhinitis^34^. Taken together, PI3K-δ may contribute to NP development through a wide range of indirect as well as direct effects on eosinophilic inflammation in the nasal cavity, thereby having potential as a useful biomarker that reflects the development of NP in CRS and possesses implications for therapeutic approaches. However, the current study was limited due to the lack of data on the exact action mechanism of PI3K-δ in chronic nasal inflammation, only showing close associations between p110δ expression in NPs and the severity of CRS. Therefore, potential mechanisms for chronic nasal inflammatory diseases including those outlined earlier should be fully investigated in the near future in various experimental systems.

In summary, for the first time, this study suggests that the PI3K-δ signaling pathway may be involved in the pathogenesis of NP, particularly eosinophilic NP associated with more severe clinical presentations and much higher radiological scores. Given the underlying heterogeneous nature of NP pathogenesis, assessing the extent of PI3K-δ activation may be used as a promising biomarker for identifying a clinically important group of CRS with NP.

## Electronic supplementary material

Supplemental Figure 1 and 2
