# Supplementary material for: Phosphoinositide 3-kinase-delta could be a biomarker for eosinophilic nasal polyps
Source: Sci Rep. 2018 Oct 30;8:15990. doi: 10.1038/s41598-018-34345-3 (PMC6207677; doi:10.1038/s41598-018-34345-3)
Supplement: Supplementary file 1 — Supplemental Figure 1 and 2 [file 41598_2018_34345_MOESM1_ESM.docx]

**[*Online data supplement*]**

**Phosphoinositide 3-kinase-delta could be a biomarker for eosinophilic nasal polyps**

**Jong Seung Kim, Jae Seok Jeong, Kyung Bae Lee, So Ri Kim, Yeong Hun Choe, Sam Hyun Kwon, Seong Ho Cho, and Yong Chul Lee**

**SUPPLEMENTARY FIGURES AND FIGURE LEGENDS**

**
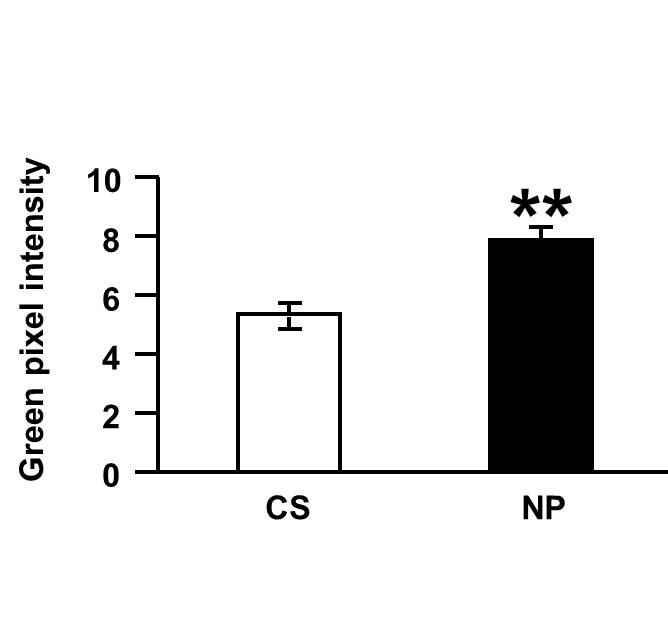
**

**Supplemental Figure 1.** Quantification of the immunofluorescence intensities for p110δ in inferior turbinate tissues from control subjects (CS) or nasal polyp (NP) tissues from chronic rhinosinusitis (CRS) patients. Bars represent mean ± SD. ^**^*P* < 0.01 versus CS.

**
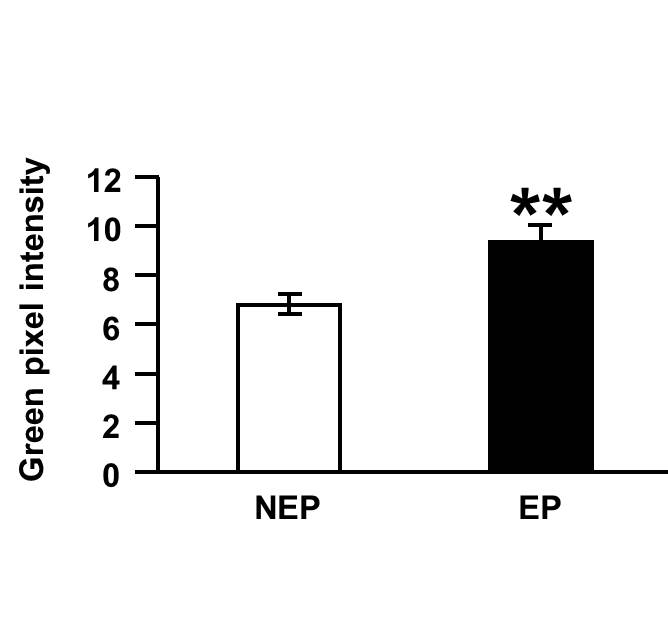
**

**Supplemental Figure 2.** Quantification of the immunofluorescence intensities for p110δ in nasal polyp (NP) tissues from chronic rhinosinusitis (CRS) patients with non-eosinophilic NP (NEP) or eosinophilic NP (EP). Bars represent mean ± SD. ^**^*P* < 0.01 versus NEP.
